# ASSOCIATION OF MMP-7 -181A>G POLYMORPHISM WITH COLORECTAL CANCER AND GASTRIC CANCER SUSCEPTIBILITY: A SYSTEMATIC REVIEW AND META-ANALYSIS

**DOI:** 10.1590/0102-672020190001e1449

**Published:** 2019-10-21

**Authors:** Mohammad ZARE, Jamal JAFARI-NEDOOSHAN, Kazem AGHILI, Hossein AHRAR, Mohammad Hossein JARAHZADEH, Neda SEIFI-SHALAMZARI, Masoud ZARE-SHEHNEH, Hossein NEAMATZADEH

**Affiliations:** 1 Shahid Sadoughi University of Medical Sciences, General Surgery;; 2 Shahid Sadoughi University of Medical Sciences, Radiology;; 3 Shahid Sadoughi University of Medical Sciences, Anesthesiology and Critical Care;; Shahrekord University of Medical Sciences, Emergency Medicine,; 5 Sadoughi University of Medical Sciences, Medical Genetics; Yazd, Yazd, Iran

**Keywords:** Matrix metalloproteinase-7, Colorectal neoplasms, Stomach neoplasms, Polymorphism, Single nucleotide, Meta-analysis, Metaloproteinase 7 da matriz, Neoplasias colorretais, Neoplasias gástricas, Polimorfismo de nucleotídeo único, Metanálise

## Abstract

**Introduction:**

The matrix metalloproteinase-7 (MMP-7) gene -181A>G polymorphism has been reported to be associated with colorectal cancer (CRC) and gastric cancer (GC) susceptibility, yet the results of these previous results have been inconsistent or controversial.

**Aim:**

To elaborate a meta-analysis to assess the association of -181A>G polymorphism of MMP-7 with CRC and GC risk.

**Methods:**

Published literature evaluating the association from PubMed, Web of Science, Google Scholar and other databases were retrieved up to April 25, 2018. Pooled odds ratio (OR) and 95% confidence interval (CI) were calculated using random- or fixed-effects model.

**Results:**

A total of 19 case-control studies, which included eleven studies on CRC (2,169 CRC cases and 2,346 controls) and eight studies on GC (1,545 GC cases and 2,366 controls) were identified. There was a significant association between MMP-7 -181A>G polymorphism and GC risk under the homozygote model (GG vs. AA: OR=1.672, 95% CI 1.161-2.409, p=0.006) and the recessive model (GG vs. GA+AA: OR=1.672, 95% CI 1.319-2.554, p=0.001), but not with CRC. By subgroup analysis based on ethnicity, an increased risk of CRC and GC was found only among Asians.

**Conclusions:**

This meta-analysis suggests that MMP-7 -181A>G polymorphisms is associated with GC risk, but not with CRC. However, our results clearly showed that the MMP-7 -181A>G polymorphism significantly increased the risk of CRC only in Asians.

## RESUMO

**Introdução:**

O polimorfismo da matriz metaloproteinase-7 (MMP-7) -181A>G tem sido relatado como associado à suscetibilidade dos cânceres colorretal (CRC) e gástrico (GC), mas os resultados desses estudos anteriores foram inconsistentes ou controversos.

**Objetivo:**

Elaborar metanálise para avaliar a associação do polimorfismo -181A> G da MMP-7 com o risco de CRC e GC.

**Métodos:**

Revisão da literatura publicada avaliando essa associação no PubMed, Web of Science, Google Acadêmico e outras bases de dados até 25 de abril de 2018. Odds ratio (OR) e o intervalo de confiança de 95% (IC) foram calculados usando dados aleatórios ou modelo de efeitos fixos.

**Resultados:**

Um total de 19 estudos caso-controle, que incluíram 11 trabalhos sobre CRC (2.169 casos de CCR e 2.346 controles) e oito sobre GC (1.545 casos de GC e 2.366 controles) foram identificados. Houve associação significativa entre o polimorfismo MMP-7 -181A>G e o risco de GC sob o modelo homozigoto (GG vs. AA: OR=1,672, IC 95% 1,161-2,409, p=0,006) e o modelo recessivo (GG vs. GA + AA: OR=1,672, IC 95% 1,319-2,554, p=0,001), mas não com CRC. Por análise de subgrupos com base na etnia, um risco aumentado de CRC e GC foi encontrado apenas entre os asiáticos.

**Conclusões:**

Esta metanálise sugere que os polimorfismos MMP-7 -181A>G estão associados ao risco de GC, mas não ao CRC. No entanto, estes resultados mostraram claramente que o polimorfismo MMP-7 -181A>G aumentou significativamente o risco de CRC apenas em asiáticos.

## INTRODUCTION

Nowadays, gastrointestinal-related cancers especially gastric cancer (GC) and colorectal cancer (CRC) are major causes of cancer-related death worldwide ^7^ ^,^ ^22^ ^,^ ^23^. Globally, CRC and GC are the third and fourth leading cause most common cancers, making up 10% and 7% of cases all new cancer cases, respectively ^23^ ^,^ ^27^. It is well-known that the development of CRC and GC can be induced by the interactions of multiple genetic and environmental factors in complex ways. However, the pathogenesis of CRC differs from that of GC in that it is affected by ethnic background, regional, life style, environmental factors and molecular pathogenesis^27^. Currently, several genes have been reported to be associated with CRC and GC, and the matrix metalloproteinases (MMPs) genes has received increasing attention^10, 29,31^.

MMPs are classified as a large family of zinc-containing proteases, which involved in normal physiological and pathological processes such as degradation and remolding of extracellular matrix, embryonic development, reproduction and cancer^6^ ^,^ ^13^. MMP-7, the smallest member of MMP family, is an endopeptidases with broad substrate specificity, which break down extracellular matrix (ECM) by degrading macromolecules including casein, type I, II, IV, and V gelatins, fibronectin, and proteoglycan^18^. In addition, MMP-7 is one of the main regulatory enzymes involved in apoptosis by releases the Fas ligand (FasL) from the membrane then induces apoptosis of neighboring cells, or decreases cancer-cell apoptosis^21^. Thus, MMP-7 promotes cell survival by resisting apoptosis through cleaving FasL. MMP7 is potentially involved in tumor metastasis and inflammatory processes^10^ ^,^ ^31^.

MMP-7 gene (also known as matrilysin) is localized on chromosome 11q21-q22 and contains 13 exons^35^. The single nucleotide polymorphism (SNP) -181A>G in promoter region of MMP-7 gene has been considered to be a candidate SNP for various conditions including gastrointestinal related malignancies^1^ ^,^ ^10^ ^,^ ^31^. There is clear evidence that MMP-7 gene up-regulation is significantly related to the promoter activity variation of the -181A>G16. Molecular epidemiological studies have reported the association of MMP-7 -181A>G polymorphism with CRC and GC risk, but the results remain conflicting rather than conclusive. Several studies previously have performed on the association of MMP-7 -181A>G polymorphism with CRC and GC risk^10^ ^,^ ^31^. However, these studies had opposite observations and additional case-control studies with larger sample sizes have been published since then. Hence, the association of MMP-7 -181A>G polymorphism remains unknown. 

Therefore, we have performed a meta-analysis of all eligible studies to derive more precise estimation of the association of MMP-7 -181A>G polymorphism with CRC and GC risk.

## METHODS

### Literature search

Following PRISMA guidance, we searched the electronic literature databases including PubMed, EMBASE, Elsevier, Science Direct, Wan Fang, Chinese National Knowledge Infrastructure (CNKI) and Chinese Biomedical Literature for all relevant articles published up to April 25, 2018. The search strategies were based on combinations of the following key words: (‘’matrix metalloproteinase-7’’ OR ‘’MMP-7’’ OR ‘’matrilysin’’ OR uterine metalloproteinase OR ‘’pump-1 protease’’ OR ‘’PUMP-1’’) AND (‘’-181A>G OR ‘’rs11568818’’) AND (‘’colorectal cancer’’ OR ‘’CRC’’) AND (‘’gastric adenocarcinoma’’ OR ‘’stomach cancer’’ OR ‘’gastric cancer’’ OR ‘’GC’’) AND (‘’gene” or ‘’allele” or ‘’genotype” or ‘’mutation” or ‘’variant” or ‘’variation” or ‘’polymorphism”), without any restriction on language. Review articles were hand-searched to find additional eligible studies and only published studies with full-text articles were included. We excluded studies that were not full-length publications articles or letters in peer-reviewed English journals. When the same patient population was included in different articles, the one with the largest population of participants or the most recent one was selected.

### Inclusion and exclusion criteria

Inclusion criteria was defined as follows: 1) published studies and contained original data; 2) case-control studies; 3) evaluating the association of MMP-7 -181A>G polymorphism with CRC and GC risk; and 4) sufficient published data available to estimate an odds ratio (OR) with 95% confidence interval (CI). Major reasons for exclusion of studies were as follows: 1) only case population, family based or linkage studies; 2) studies that could not offer the number of cases and controls or other essential data; 3) reviews, abstracts, letters to editor, case reports or animal studies; 4) duplicate of previous publication or studies with overlapping patient populations; and 5) studies without histologically confirmed of CRC and GC. For more than two studies with overlapping data, the study with the most subjects or newest published data was selected.

### Data extraction

Data were carefully extracted from all eligible publications by two of the authors independently. If the study provided stratum information, the data coming from similar stratum were added up to make full use of the data. Disagreements between the two authors were resolved by discussing the results with a third one. For each study, the following variables were collected: first author’s name, year of publication, country, ethnicity of participants, number of cases and controls, genotyping methods, and allele numbers and genotype distributions in cases and controls, minor allele frequencies (MAFs) in control subjects, and the results of Hardy-Weinberg equilibrium (HWE) test. Different ethnicities were categorized as Asian, Caucasian and Latinos (mixed). Study designs were stratified to population-based studies and hospital-based studies.

### Statistical analysis

The strength of the association of MMP-7 -181A>G polymorphism with CRC and GC risk was measured using odds ratios (ORs) with 95% confidence intervals (CIs). The statistical significance of the pooled OR was assessed with the Z-test and p<0.05 was considered significant. The pooled ORs were performed under five genetic models, i.e., allele (G vs. A), heterozygote (GA vs. AA), homozygote (GG vs. AA), dominant (GG+GA vs. AA) and recessive (GG vs. GA+AA). The between-study heterogeneity was evaluated by a chi-square-based Q test, which p value for the Q-test less than 0.10 indicates existing heterogeneity among studies. In addition, the I^2^ statistics was used to quantify the proportion of the total variation across studies due to heterogeneity. A high value of I2 indicated a higher probability of the existence of heterogeneity (I^2^=0% to 25%, no heterogeneity; I^2^=25% to 50%, moderate heterogeneity; I^2^=50% to 75%, large heterogeneity; and I^2^=75% to 100%, extreme heterogeneity). A random-effects (DerSimonian- Laird method) or fixed-effects (Mantel-Haenszel method) model was used to calculate pooled effect estimates in the presence or absence of heterogeneity. HWE of genotype distribution in the controls of included studies was conducted using by Pearson’s x ^2^ test, in which p-value less than 0.05 was considered significantly deviating from HWE. Subgroup analyses were performed by ethnicity, genotyping method, HWE status, source of controls and cancer type (CRC and GC). In addition, to consider the possible sources of heterogeneity, the studies we stratified. To validate the reliability of the results, sensitivity analysis was performed though omitting one case-control study each time, as well as limiting this meta-analysis to studies which were conformed to HWE. Funnel plots and Egger’s linear regression test were used to diagnose potential publication bias (p<0.05). All analyses were performed with the comprehensive meta-analysis (CMA) 2.0 software (Biostat, USA). Two-sided p-values<0.05 were considered statistically significant.

## RESULTS

### Extraction process and study characteristics

**FIGURE 1:**
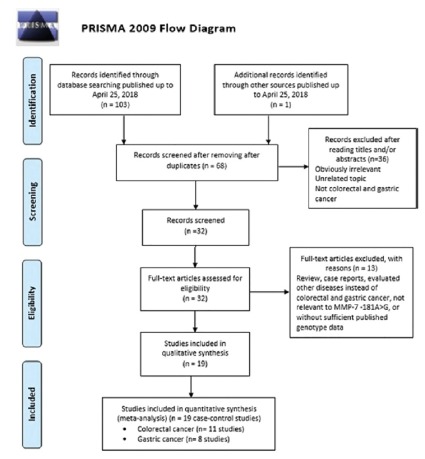
The study selection and inclusion process.

The flow diagram of literature search was given in Figure 1. The initial search of databases yielded 103 relevant publications based on our literature search strategy, and an additional one study was identified through hand searching. However, 36 of them were ruled out because of duplicate results obtained from multiple databases, 68 articles remained. In addition, after the titles and abstracts of the 68 articles were reviewed, 49 full-text irrelevant studies were excluded. Finally, 19 eligible case-control studies with 3,714 cases and 4,712 controls were included in this meta-analysis. The characteristics of studies included in the current meta-analysis are shown in Table 1. Among these studies, eleven studies with 2,169 CRC cases and 2,346 controls were on CRC^2^ ^-^ ^4^ ^,^ ^8^ ^,^ ^16^ ^,^ ^19^ ^,^ ^20^ ^,^ ^24^ ^,^ ^28^ ^,^ ^33^, and eight studies with 1,545 GC cases and 2,366 controls were on GC^5^ ^,^ ^11^ ^,^ ^12^ ^,^ ^14^ ^,^ ^15^ ^,^ ^17^ ^,^ ^26^ ^,^ ^34^. By ethnics, there were 13 studies of Asians (countries: Korea, Japan, China, Iran, Kashmir, Taiwan, and India), four studies of Caucasians (countries: Italy, France, Poland and Netherland), and two studies of Latinos (countries: Brazil and Mexico). According to the control source, ten studies were hospital-based, eight studies were population-based and one study was not clear. The studies used four different genotyping methods including direct sequencing, TaqMan, polymerase chain reaction-restriction fragment length polymorphism (PCR-RFLP) analysis and tetra-primer amplification refractory mutation system-polymerase chain (ARMS-PCR). All of the studies indicated that the distribution of genotypes in the controls was consistent with HWE except for two studies (Table 1).

**TABLE 1:** Main characteristics of all studies included in the meta-analysis

First Author	Country (Ethnicity)	Genotyping Technique	SOC	Case/Control	Cases			Controls					MAFs		HWE	
					Genotype			Allele		Genotype			Allele			
					AA	AG	GG	A	G	AA	AG	GG	A	G		
Colorectal Cancer																
Ghilardi 2003	Italy (Caucasian)	Sequencing	PB	58/111	15	28	15	58	58	36	61	14	133	89	0.400	0.129
Lievre 2006	France (Caucasian)	TaqMan	PB	596/565	191	272	131	658	534	187	259	119	1083	497	0.439	0.097
Woo 2007	Korea (Asian)	PCR-RFLP	PB	185/304	163	22	0	348	22	265	39	0	565	39	0.064	0.232
Ohtani 2009	Japan (Asian)	PCR-RFLP	HB	119/67	110	9	0	229	9	55	12	0	122	12	0.089	0.420
de Lima 2009	Brazil (Latinos)	PCR-RFLP	HB	108/113	36	56	16	128	88	41	57	15	139	87	0.384	0.487
Fang 2010	China (Asian)	PCR-RFLP	PB	252/237	22	30	0	474	30	218	19	0	455	19	0.040	0.520
Dziki 2011	Poland (Caucasian)	PCR-RFLP	HB	184/205	99	93	52	171	197	66	94	45	216	194	0.473	0.294
Moreno-Ortiz 2014	México (Latinos)	PCR-RFLP	HB	102/121	46	51	5	143	61	49	54	18	152	90	0.371	0.622
Motoval-Bashi 2015	Iran (Asian)	ARMS-PCR	NS	61/77	11	31	19	53	69	6	40	31	52	102	0.708	0.156
Banday 2016	Kashmir (Asian)	PCR-RFLP	HB	142/184	43	82	13	176	108	61	84	39	206	162	0.440	0.317
Yueh 2018	Taiwan (Asian)	PCR-RFLP	HB	362/362	318	38	6	674	50	311	43	8	665	59	0.081	=0.001
Gastric Cancer																
Zhang 2005	China (Asian)	PCR-RFLP	PB	201/350	167	34	0	368	34	316	33	1	662	35	0.050	0.888
Kubben 2006	Nederland (Caucasian)	PCR-RFLP	PB	79/169	34	37	8	105	53	46	106	17	198	140	0.414	=0.001
Sugimoto 2008	Japan (Asian)	PCR-RFLP	HB	160/434	133	27	0	293	27	393	40	1	826	42	0.048	0.986
Li 2008	China (Asian)	PCR-RFLP	PB	338/380	280	56	2	616	60	342	37	1	721	39	0.051	0.999
Kim 2011	Korea (Asian)	PCR-RFLP	HB	153/326	128	24	1	280	26	280	45	1	605	47	0.072	0.565
Malik 2011	India (Asian)	PCR-RFLP	PB	108/195	29	39	40	98	119	63	92	40	218	172	0.441	0.547
Fang 2013	China (Asian)	PCR-RFLP	HB	246/252	236	10	0	482	10	222	30	0	474	30	0.059	0.315
Kesh 2015	India (Asian)	PCR-RFLP	HB	260/260	107	108	45	322	198	118	116	26	352	168	0.323	0.746

PCR-RFLP=polymerase chain reaction-restriction fragment length polymorphism; ARMS-PCR=tetra-primer amplification refractory mutation system-polymerase chain; SOC=source of control; HB=hospital-based; PB=population-based; MAF=minor allele frequency; HWE=Hardy-Weinberg equilibrium; NS=not stated

### Quantitative synthesis

#### Overall study

**FIGURE 2:**
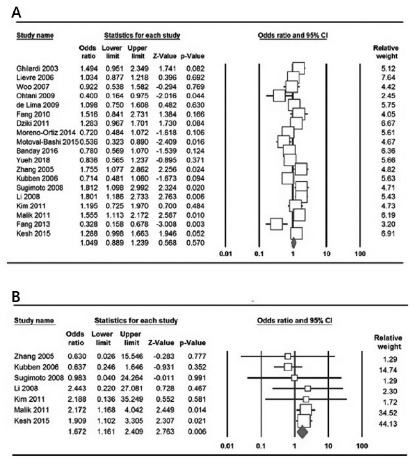
Forest plot for the association of MMP-7 -181A>G polymorphism with CRC and GC risk: A) the allele model (G vs. A) in overall estimations; B) the homozygote model (GG vs. AA) in GC studies

Table 2 listed the main results of the meta-analysis of MMP-7 -181A>G polymorphism with CRC and GC risk. We pooled all the 19 case-control studies together to assess the overall association of MMP-7 -181A>G polymorphism with CRC and GC risk. Overall, no significant main effects on CRC and GC susceptibility were observed in the overall population under all the five genetic models, i.e., allele (G vs. A: OR=1.049, 95% CI 0.889-1.239, p=0.570, Figure 2A), heterozygote (GA vs. AA: OR=1.083, 95% CI 0.813-1.443, p=0.586), homozygote (GG vs. AA: OR=0.982, 95% CI 0.701-1.375, p=0.915), dominant (GG+GA vs. AA: OR=1.061, 95% CI 0.869-1.296, p=559) and recessive (GG vs. GA+AA: OR=1.084, 95% CI 0.786-1.495, p=0.622).

**TABLE 2:** Meta-analysis for the association of MMP-7 -181A>G polymorphism with CRC and GC risk

Subgroup	Genetic model	Type of model	Heterogeneity		Odds ratio (OR)		Publication bias			
			I2 (%)	PH	OR	95% CI	ZOR	POR	PBeggs	PEggers
Overall (n=19)	G vs. A	Random	70.90	=0.001	1.049	0.889-1.239	0.568	0.570	0.293	0.483
	GA vs. AA	Random	82.36	=0.001	1.083	0.813-1.443	0.545	0.586	0.293	0.906
	GG vs. AA	Random	54.65	0.006	0.982	0.701-1.375	-0.107	0.915	0.766	0.611
	GG+GA vs. AA	Random	65.43	=0.001	1.061	0.869-1.296	0.585	0.559	0.068	0.223
	GG vs. GA+AA	Random	60.48	0.001	1.084	0.786-1.495	0.494	0.622	0.692	0.651
Colorectal cancer (n=11)	G vs. A	Random	58.32	0.008	0.947	0.791-1.133	-0.593	0.553	0.275	0.345
	GA vs. AA	Random	85.27	=0.001	1.101	0.721-1.682	0.444	0.657	0.876	0.779
	GG vs. AA	Random	58.51	0.018	0.791	0.529-1.183	-1.142	0.253	0.536	0.330
	GG+GA vs. AA	Fixed	42.24	0.068	1.032	0.894-1.192	0.431	0.667	0.533	0.355
	GG vs. GA+AA	Random	68.09	0.003	0.878	0.589-1.309	-0.639	0.523	0.901	0.401
Gastric cancer (n=8)	G vs. A	Random	76.10	=0.001	1.215	0.897-1.645	1.256	0.209	0.901	0.459
	GA vs. AA	Random	79.18	=0.001	1.063	0.711-1.590	0.300	0.764	0.035	0.233
	GG vs. AA	Fixed	0.00	0.485	1.672	1.161-2.409	2.763	0.006	0.229	0.499
	GG+GA vs. AA	Random	78.48	=0.001	1.132	0.771-1.660	0.632	0.527	0.107	0.222
	GG vs. GA+AA	Fixed	0.00	0.789	1.835	1.319-2.554	3.604	=0.001	0.367	0.310
Colorectal Cancer										
Asian (n=6)	G vs. A	Fixed	47.83	0.088	0.798	0.661-0.964	-2.337	0.019	0.707	0.796
	GA vs. AA	Random	91.80	=0.001	1.216	0.486-3.040	0.416	0.676	0.707	0.959
	GG vs. AA	Fixed	0.00	0.612	0.490	0.286-0.838	-2.606	0.009	1.000	0.995
	GG+GA vs. AA	Fixed	46.52	0.096	0.896	0.708-1.134	-0.915	0.360	0.707	0.189
	GG vs. GA+AA	Fixed	0.00	0.397	0.530	0.340-0.826	-2.808	0.005	1.000	0.587
Caucasian (n=3)	G vs. A	Fixed	40.68	0.185	1.123	0.981-1.285	1.676	0.094	0.296	0.126
	GA vs. AA	Fixed	38.97	0.194	0.923	0.744-1.143	-0.736	0.461	1.000	0.844
	GG vs. AA	Fixed	59.28	0.086	1.054	0.812-1.366	0.393	0.694	1.000	0.643
	GG+GA vs. AA	Fixed	53.13	0.118	1.180	0.960-1.451	1.569	0.117	1.000	0.460
	GG vs. GA+AA	Fixed	51.03	0.130	1.210	0.962-1.522	1.628	0.104	0.296	0.001
Latinos (n=2)	G vs. A	Fixed	55.62	0.133	0.897	0.681-1.181	-0.773	0.440	NA	NA
	GA vs. AA	Fixed	0.00	0.795	1.059	0.709-1.581	0.278	0.781	NA	NA
	GG vs. AA	Random	75.98	0.041	0.625	0.157-2.488	-0.667	0.505	NA	NA
	GG+GA vs. AA	Fixed	0.00	0.476	0.947	0.645-1.392	-0.276	0.783	NA	NA
	GG vs. GA+AA	Random	76.58	0.039	0.606	0.162-2.267	-0.743	0.457	NA	NA
Gastric Cancer										
Asian (n=7)	G vs. A	Random	69.58	0.003	1.331	0.994-1.782	1.918	0.055	1.000	0.451
	GA vs. AA	Random	74.59	0.001	1.197	0.815-1.758	0.917	0.359	0.229	0.368
	GG vs. AA	Fixed	0.00	0.976	1.976	1.331-2.934	3.380	0.001	0.259	0.298
	GG+GA vs. AA	Random	71.98	0.002	1.281	0.898-1.826	1.367	0.172	0.367	0.341
	GG vs. GA+AA	Fixed	0.00	0.953	2.022	1.416-2.886	3.877	=0.001	0.259	0.240

NA=not applicable

### Colorectal cancer

Table 2 also listed the main results of the meta-analysis of MMP-7 -181A>G polymorphism with CRC risk. When all the 11 eligible studies were pooled into the meta-analysis of MMP-7 -181A>G polymorphism, we have not found evidence of a significant MMP-7 -181A>G polymorphism with CRC risk under all the five genetic models. In the subgroup analysis by ethnicity, significantly increased risk of CRC was observed in Asians under three genetic models, i.e., allele (G vs. A: OR=0.798, 95% CI 0.661-0.964, p=0.019, Figure 2A), homozygote (GG vs. AA: OR=0.490, 95% CI 00.286-0.838, p=0.009) and recessive model (GG vs. GA+AA: OR=0.530, 95% CI 0.340-0.826, p=0.005), but not in Caucasians and Latinos populations (Table 2).

We also performed subgroup analyses based on the source of control and genotyping method, when it was available (Table 3). The hospital-based subgroup analysis revealed that the presence of the MMP-7 -181A>G polymorphism was related to a higher risk of CRC under the homozygote model (GG vs. AA: OR=0.671, 95% CI 0.484-0.951, p=0.023). In the PCR-RFLP group, significantly increased association between MMP-7 -181A>G polymorphism and CRC risk was found under the homozygote model (GG vs. AA: OR=0.680, 95% CI 0.486-0.950, p=0.024, Table 3).

**TABLE 3:** Meta-analysis for the association of MMP-7 -181A>G polymorphism with CRC and GC risk

Subgroup	Genetic model	Type of model	Heterogeneity		Odds ratio (OR)				Publication bias	
			I2 (%)	PH	OR	95% CI	ZOR	POR	PBeggs	PEggers
Colorectal Cancer										
PB (n=4)	G vs. A	Fixed	21.99	0.279	1.089	0.943-1.257	1.159	0.246	0.734	0.390
	GA vs. AA	Random	94.02	=0.001	1.961	0.660-5.829	1.211	0.226	0.308	0.456
	GG vs. AA	Fixed	65.72	0.088	1.178	0.871-1.595	1.063	0.288	NA	NA
	GG+GA vs. AA	Fixed	0.00	0.518	1.089	0.890-1.333	0.829	0.407	0.308	0.414
	GG vs. GA+AA	Fixed	71.99	0.059	1.153	0.885-1.503	1.054	0.292	NA	NA
HB (n=6)	G vs. A	Random	59.11	0.032	0.884	0.688-1.135	-0.969	0.333	0.060	0.118
	GA vs. AA	Fixed	45.41	0.103	0.892	0.720-1.106	-1.039	0.299	1.000	0.585
	GG vs. AA	Fixed	25.38	0.250	0.671	0.484-0.951	-2.260	0.023	0.806	0.583
	GG+GA vs. AA	Random	56.09	0.044	0.971	0.701-1.347	-0.174	0.862	0.707	0.192
	GG vs. GA+AA	Random	73.18	0.005	0.702	0.367-1.341	-1.072	0.284	0.806	0.258
PCR-RFLP (n=8)	G vs. A	Random	52.42	0.040	0.934	0.751-1.160	-0.620	0.536	0.386	0.396
	GA vs. AA	Random	89.23	=0.001	1.212	0.665-2.210	0.627	0.531	0.386	0.423
	GG vs. AA	Fixed	25.41	0.252	0.680	0.486-0.950	-2.261	0.024	0.806	0.583
	GG+GA vs. AA	Fixed	47.45	0.065	1.022	0.785-1.331	0.165	0.869	0.901	0.260
	GG vs. GA+AA	Random	73.18	0.005	0.702	0.367-1.341	-1.072	0.284	0.806	0.258
Gastric Cancer										
PB (n=4)	G vs. A	Random	77.98	0.003	1.360	0.884-2.093	1.401	0.161	0.734	0.873
	GA vs. AA	Random	83.08	=0.001	1.138	0.601-2.155	0.397	0.692	0.089	0.192
	GG vs. AA	Fixed	39.16	0.177	1.504	0.911-2.484	1.595	0.111	0.734	0.678
	GG+GA vs. AA	Random	81.36	0.001	1.244	0.693-2.233	0.733	0.464	0.089	0.239
	GG vs. GA+AA	Fixed	0.00	0.400	1.819	1.173-2.819	2.673	0.008	0.734	0.490
HB (n=4)	G vs. A	Random	80.17	0.002	1.048	0.622-1.766	0.178	0.859	0.734	0.478
	GA vs. AA	Random	80.40	0.002	0.985	0.545-1.781	-0.049	0.961	0.734	0.576
	GG vs. AA	Fixed	0.00	0.918	1.884	1.107-3.204	2.336	0.020	0.296	0.644
	GG+GA vs. AA	Random	81.01	0.001	1.016	0.564-1.831	0.054	0.957	0.308	0.475
	GG vs. GA+AA	Fixed	0.00	0.901	1.858	1.124-3.069	2.417	0.016	0.296	0.621

PB=population-based; HB=hospital-based; PCR-RFLP=polymerase chain reaction-restriction fragment length polymorphism; NA= not applicable

### Gastric cancer

Table 2 also listed the main results of the meta-analysis of MMP-7 -181A>G polymorphism with GC risk. There was a significant association between MMP-7 -181A>G polymorphism and GC risk under two genetic models, i.e., homozygote (GG vs. AA: OR=1.672, 95% CI 1.161-2.409, p=0.006, Fig 2B) and recessive (GG vs. GA+AA: OR=1.835, 95% CI 1.319-2.554, p=0.001). Similarly, when stratified by ethnicity, a significant association between MMP-7 -181A>G polymorphism and increased risk of GC was detected among Asians under the homozygote model (GG vs. AA: OR=1.975, 95% CI 1.331-2.934, p=0.006) and the recessive model (GG vs. GA+AA: OR=2.022, 95% CI 1.416-2.886, p=0.001).

The studies were further stratified on the basis of source of controls (Table 3). When stratifying by source of control, a significant association between MMP-7 -181A>G polymorphism and increased risk of GC was detected in population-based studies under the recessive model (GG vs. GA+AA: OR=1.819, 95% CI 1.173-2.819, p=0.008), and in hospital-based studies under two genetic models, i.e., homozygote (GG vs. AA: OR=1.884, 95% CI 1.107-3.204, p=0.020) and recessive (GG vs. GA+AA: OR=1.858, 95% CI 1.124-3.069, p=0.016).

### Heterogeneity analysis

Heterogeneity was detected among studies under all the five genetic models, i.e., allele (G vs. A: I2=70.90%, PH=0.001), heterozygote (GA vs. AA: I2=82.36%, PH=0.001), homozygote (GG vs. AA: I2=54.65%, PH=0.006), dominant (GG+GA vs. AA: I2=65.43%, PH=0.001) and recessive (GG vs. GA+AA: I2=65.4%, PH=0.001). Thus, to explore the potential sources of heterogeneity across studies, we assessed the pooled ORs via stratification by cancer type, ethnicity, genotyping method, HWE status and source of controls. The results showed that the heterogeneity effectively removed by subgroup analyses based on ethnicity among studies on CRC. Therefore, we found that genotyping method, HWE status and source of controls did not contribute to substantial heterogeneity among the meta-analysis.

### Sensitivity analysis

To evaluate the effect of individual study on the pooled ORs and stability of the meta-analysis results, we excluded one study at a time. However, the omission of any single study made no significant difference, suggesting that the results of this meta-analysis were stable. Moreover, sensitivity analysis was performed after excluding HWE-violating studies, and the corresponding pooled ORs were not qualitatively altered (data not shown).

### Publication bias

**FIGURE 3:**
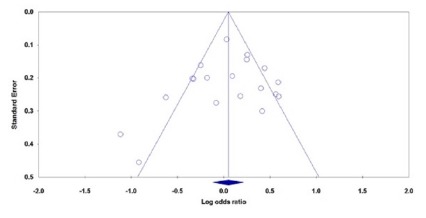
Begg’s funnel plots of the MMP-7 -181A>G polymorphism with CRC and GC risk for publication bias test under the allele model (G vs. A): each point represents a separate study for the indicated association

Both Begg’s funnel plot and Egger’s test were performed to assess the publication bias of literature. Begg’s funnel plots did not reveal any evidence of obvious asymmetry under all five genetic models in the overall meta-analysis. For example, the shape of the funnel plot did not indicate any evidence of obvious asymmetry under the allele model (Figure 3), and the Egger’s test suggested the absence of publication bias (PBeggs=0.293 and PEggers=0.483). However, the results of Egger’s regression test showed evidence of publication bias among Caucasian’s studies on CRC under the recessive model (GG vs. GA+AA: PBegg’s=0.296, PEggers=0.001).

### Minor allele frequencies (MAFs)

The minor allele frequencies (MAFs) of the MMP-7 -181A>G polymorphism are shown in Table 2. The allele and genotype distributions of MMP-7 -181A>G polymorphism exhibited ethnic variations. The MMP-7 -181A>G polymorphism MAF in overall populations, Asians, Caucasians and Latinos were 37.4% (4.0%-70.8%), 37.4% (4.0%-70.8%), 43.65% (40.0%-47.30%), and 37.75% (37.1%-38.4%), respectively.

## DISCUSSION

MMP-7 gene is implicated in cancer susceptibility and metastasis in a variety of gastrointestinal-related cancers ^19, 11^. In the recent decade, several molecular epidemiological studies have been performed to assess the association of MMP-7 -181A>G polymorphism with CRC and GC risk. However, the results were conflicting. Thus, we conducted a comprehensive meta-analysis involving published data, to assess the strength of association of MMP-7 -181A>G polymorphism CRC and GC risk. In current meta-analysis, a total of 19 case-control studies including 3,714 cases and 4,712 controls were recruited. The subgroup analysis by cancer type, showed that there was significant association between the MMP-7 -181A>G polymorphism and increased risk of GC in overall estimations, but not with CRC. In a stratified analysis by ethnicity, our results indicated that MMP-7 -181A>G polymorphism was associated with a significantly increased risk of CRC and GC in Asians. Moreover, stratified analysis according to source of controls and genotyping method revealed a significantly increased risk of CRC and GC in participants with the MP-7 -181A>G polymorphism in those studies involving PCR-RFLP, population-based and hospital-based (Table 3).

Our results were consistent with the previous meta-analyses on MMP-7 -181A>G polymorphism and GC risk. Thus, MMP-7 -181A>G polymorphism might serve as a susceptibility marker to GC risk. However, our results are inconsistent with the previous meta-analyses on MMP-7 -181A>G polymorphism and CRC risk. In 2013, two meta-analyses by Ke et al and Yang et al were conducted to examine the association between MMP-7 -181A>G polymorphism and CRC risk ^10^ ^,^ ^31^. Ke et al in meta-analysis of seven case-control have found a significant association between MMP-7 -181A>G polymorphism and CRC under the homozygote model (GG vs. AA, OR=1.13, 95% CI=1.01-1.26) ^10^. Similarly, Yang et al in a meta-analysis of seven studies with 1,502 CRC cases and 1,602 controls found significant association between MMP-7 -181A>G polymorphism and CRC under the homozygote model (OR=1.31, 95% CI 1.02-1.69) ^31^. However, their findings about MMP-7 -181A>G polymorphism and CRC risk essentially remains an open field, as the number of studies is considerably smaller than that needed to yield a robust conclusion. In addition, the previous meta-analyses did not perform stratified analysis by ethnicity to identify possible association of MMP-7 -181A>G polymorphism with CRC among different ethnic groups.

Between-studies heterogeneity plays an important role when performing a meta-analysis ^32^. Heterogeneity could result from study design, genotyping error, selection bias, population stratification, sample size, allelic heterogeneity, or chance ^9^ ^,^ ^25^. Therefore, finding the source of heterogeneity is very important for the final result of meta-analysis. Through performing sub group analysis, we found that the heterogeneity could not be explained by genotyping method, HWE status and source of controls in this meta-analysis. However, the results showed that the heterogeneity effectively removed by subgroup analysis based on ethnicity among studies on CRC, indicating that studies among Asian populations regarding CRC might be a source of the heterogeneity in our meta-analysis.

This meta-analysis had three main strengths. First, this is the biggest and most recent meta-analysis of the association of MMP-7 -181A>G polymorphism with CRC and GC risk, and it was more powerful than previous single case-control studies. Second, this is the first meta-analysis by subgroup analysis showed that the MMP-7 -181A>G polymorphism was associated with CRC risk in Asians. Third, a comprehensive searching strategy from several electronic databases with manual search made the eligible studies included as much as possible.

Despite the clear strengths of this meta-analysis, limitations of our meta-analysis should be noted. First, although all the eligible studies were included to this meta-analysis, the sample size of the included studies was not large enough, which could increase the likelihood of type I and type II errors. Therefore, there was a lack of statistical power to better evaluate the association of MMP-7 -181A>G polymorphism with CRC and GC risk. Second, most of included studies in the present meta-analysis mainly provided data in Asians. In addition, the sample size was relatively small for stratified analyses by ethnicity and might not have provided sufficient power to estimate the association of MMP-7 -181A>G polymorphism among different ethnic groups. Third, although the funnel plot and Egger’s test did not show evidence of publication bias in overall estimations, the influence of bias in the present analysis could not be completely excluded. For example, the negative findings are usually difficult to get published, or in this meta-analysis we have included only studies published in English, which produced selection bias at the start of our study. Fourth, the present meta-analysis was based primarily on unadjusted effect estimates, because most studies did not provide the adjusted OR and 95%CI controlling for potential confounding factors, thus the effect estimates were relatively imprecise. If individual data were available, adjusted ORs could be obtained to give a more precise analysis. Finally, it is well known that CRC and GC are multifactor conditions; however, the effects of gene-gene and gene-environment interactions were not addressed in the current meta-analysis.

## CONCLUSION

This meta-analysis indicated that the MMP-7 -181A>G polymorphism might be a risk factor for susceptibility to GC in overall estimations and in Asians. However, our results clearly showed that the MMP-7 -181A>G polymorphism significantly increased the risk of CRC only in Asians. Considering the limited sample size and ethnicities, well-designed studies taking into consideration of gene-gene and gene-environment interactions should be performed to confirm our results.
